# Efficacy and Safety of the Combination Treatment of Rituximab and Dexamethasone for Adults with Primary Immune Thrombocytopenia (ITP): A Meta-Analysis

**DOI:** 10.1155/2018/1316096

**Published:** 2018-12-12

**Authors:** Jia Wang, Ya Li, Chong Wang, Yayue Zhang, Chong Gao, Haiyan Lang, Xinyi Chen

**Affiliations:** ^1^Department of Oncology and Hematology, Dongzhimen Hospital, Beijing University of Chinese Medicine, Beijing, China; ^2^Beijing Hospital of Traditional Chinese Medicine, Clinical Medical College of Traditional Chinese Medicine, Capital Medical University, Beijing, China; ^3^Department of Hematology, Dongfang Hospital, Beijing University of Chinese Medicine, Beijing, China

## Abstract

*Objective. *To conduct a meta-analysis, assessing the efficacy and safety of the combination treatment of dexamethasone and rituximab for adults with ITP (primary immune thrombocytopenia).* Methods. *Randomized controlled trials that compared rituximab and dexamethasone combination treatment to dexamethasone monotherapy in the treatment of adults with ITP were collected by searching Pubmed, Embase, Cochrane, China National Knowledge (CNKI), Wanfang database, and Sino Med. We conducted pooled analyses on OR (overall response) rate, CR (complete response) rate, PR (partial response) rate, SR (sustained response) rate, R (relapse) rate, change in Treg cell count (mean [SD]), and AE (adverse event). GRADE pro scale was used to assess the quality of the evidence. Publication bias was assessed with Egger's test method.* Results. *A total of 11 randomized controlled trials were eligible for inclusion. The overall efficacy estimates favored combination arm in terms of OR rate at month 3, CR rate at week 4 and month 3, SR rate, and Treg cell count at week 2. Subgroup analysis showed that females obtained a higher OR rate than males did at week 4. No significant difference was found in pooled analysis of relapse rate between combination arm and monotherapy arm. The comparison of serious AE and other AEs showed no significant difference either. A total of 19 outcomes were assessed by GRADE pro software, of which 79% (15/19) was scaled as moderate-to-high level. Publication bias existed in studies on OR at week 4 (*P*=0.025), CR at week 4 (*P*=0.017), infection (*P*=0.006), and rash (*P*=0.028) of the AEs.* Conclusion. *Dexamethasone combined with rituximab can provide a better long-term response in the treatment of adults with ITP and will not increase the risk of adverse effects.

## 1. Introduction

Primary immune thrombocytopenia (ITP) is well known as an autoimmune disease, which is characterized by immune-mediated peripheral platelet destruction and impaired platelet production in the bone marrow with a consequent increased tendency of bleeding. To date, the pathogeneses of ITP are thought to involve phagocytosis and platelet destruction in the spleen mediated by autoantibodies against platelet-specific antigens and impaired megakaryocyte maturation which can reduce the production of new platelet [1]. 

The platelet threshold of<30×10^9^/L is widely determined as a trigger for treatment, in which corticosteroid is most frequently used as the first-line treatment [2, 3]. However, less than half of the patients with ITP can be ‘cured' by first-line therapy, and many of them require second-line treatments [4]. Rituximab (RTX), a human/murine monoclonal antibody targeting CD20 B-cell surface antigen, can regulate the miscellaneous autoimmune disorders by B-cell depletion [5]. It was reported that rituximab alone can provide an approximately 20% to 40% long-term response rate [6, 7]. Additionally, rituximab has been shown to increase Treg cell count of patients with ITP [8–10]. Given that a patient may have a contraindication to a specific monotherapy, adverse events, and a limited long-term response, combination treatment is more and more preferred by clinicians in the treatment with ITP. The combination of rituximab and corticosteroid has potential benefit, which might enhance the efficacy by a direct effect on blood vessels, together with a regulation effect on both humoral and cellular immune disorder. A recent RCT showed that, for newly diagnosed ITP, dexamethasone combining rituximab provided a sustained response rate of 58% at 6 months, which was significantly higher than that of dexamethasone alone (37%) [11]. This result was consistent with that of an earlier study, in which the SR rate of combination arm was much higher than that of dexamethasone arm (63% vs 36%) [12].

However, it might be difficult for a single study to provide sufficient data on its own to confirm the efficacy of the combination treatment of rituximab and corticosteroid. What is more, it still needs to be verified whether the combination treatment will increase the incidence of adverse events. Therefore, we performed this meta-analysis of randomized controlled trials (RCTs) to clarify the efficacy and safety of the combination treatment of rituximab and corticosteroid for adults with ITP.

## 2. Methods

### 2.1. Data Sources and Search Strategy

This meta-analysis was reported in accordance with PRISMA (Preferred Reporting Items for Systematic reviews and Meta-Analyses) Statement. We collected relevant studies published from inception to Apr 29, 2018, by searching Pubmed, Embase, Cochrane, China National Knowledge (CNKI), Wanfang database, and Sino Med. The search strategy was made according to PICOS (Population, Intervention, Comparison, Outcome, and Study design) principle. Medical Subject Heading (MeSH) terms and key words were as follows: (idiopathic thrombocytopenic purpura OR Immune Thrombocytopenic Purpura OR Werlhof 's Disease) AND (rituximab OR CD20 Antibody) AND (dexamethasone OR hexadecadrol OR dexasone) AND (randomized controlled trial). Manual search was also conducted by the key words above for all potentially eligible studies. Searches were restricted to articles published in English or Chinese.

### 2.2. Study Selection

Studies that were randomized controlled trials done in adults with ITP, compared rituximab and dexamethasone combination treatment (RTX+DXM) to dexamethasone monotherapy (DXM), had at least 28 days duration of intervention, and reported OR (overall response) rate, CR (complete response) rate, PR (partial response) rate, SR (sustained response rate) rate, R (relapse) rate, change in Treg cell count (mean[SD]), or AE (adverse event), were included.

Exclusion criteria were as follows: (1) reviews, case report, and irrelevant topics; (2) studies without control arms (dexamethasone monotherapy); (3) studies on secondary ITP; (4) studies that included participants<18 years old.

Studies that had 3 arms were also included if the data of RTX+DXM arm and DXM arm were extractable from the articles. If duplicate publication were found, only the latest and most complete one was included.

### 2.3. Data Extraction

Two investigators (YL and CW) reviewed titles and abstracts of all articles independently. Studies that met our inclusion criteria were retrieved for full-text reading. Two independent investigators (YL and CG) conducted the detailed extraction and analyses of data; discrepancies were resolved by a third investigator (HYL).

We compared combination treatment of rituximab and dexamethasone to dexamethasone monotherapy, and no treatment history was restricted. The outcomes assessed were as follows: response rate; relapse rate during or after the intervention; Treg cell count before and after the intervention; adverse events during the intervention. The following detailed data were extracted from each included study: total number of participants, baseline demographics (age, gender), therapeutic regimen; number of participants achieving OR, CR, and PR at week 4 or month 3 (OR=CR+PR), number of participants achieving SR at 6 months or 12 months; number of participants who relapsed during or after the intervention; Treg cell count (mean[SD]) before or at weeks 2 and 4 of intervention; number of participants experiencing serious AE or regular AE (infection, hyperglycemia, hypertension, electrolyte disorder, fever, and hypersensitivity).

### 2.4. Quality Assessment and Risk of Bias

All of the eleven selected studies were randomized controlled trials. GRADE pro scale (Grading of Recommendations Assessment, Development and Evaluation) was used to assess the quality of the evidence, which was scaled as high, moderate, low, or very low. Risk of bias was determined according to Cochrane Handbook of Systematic Reviews of Interventions.

### 2.5. Data Analysis

Here we assessed the efficacy of combination treatment (RTX+DXM) by following outcomes: achievement of OR, CR, and PR at week 4 or month 3 (OR=CR+PR); achievement of SR at month 6 or month 12; incidence of relapse; change in Treg cell count. Definitions of CR were consistent among the selected studies (platelet count≥100×10^9^/L) [13]. PR was determined as platelet count≥30×10^9^/L in most selected studies [10, 14–19]. Consequently, we chose to include PR as an outcome and considered achievement of OR as total number of participants achieving CR and PR. SR was defined as platelet count≥50×10^9^/L at month 6 [8, 11, 12, 17, 19]. Definition of relapse was dropping in platelet count to≤30×10^9^/L following a CR or PR[8, 10–12, 20].

The safety of combination treatment (RTX+DXM) was assessed by incidence of serious AEs or other AEs. We planned to report whether the combination treatment will increase the incidence of adverse effects in monotherapy. Considering the most frequent adverse effects reported by the selected studies, we compared the safety of combination treatment (RTX+DXM) to monotherapy (DXM) by the number of participants who experienced following AEs: serious AE, infection, hyperglycemia, hypertension, electrolyte disorder, fever, and rash. Serious AE was determined according to National Cancer Institute Common Toxicity Criteria for Adverse Events (CTCAE) [8, 11, 12].

All of the event rates were analyzed as dichotomous variables. Relative risk ratio (RR) was obtained by using random-effects model (DerSimonian-Laird method). Fixed-effect model was applied to the analysis when heterogeneity did not exist. We analyzed Treg cell count before and after 2 and 4 weeks of intervention as continuous variables. To account for expected heterogeneity, pooled estimates of mean difference (MD) were calculated by using random-effects model.

We assessed the risk of publication bias by constructing a funnel plot of each RCT' s effect size. Asymmetry of each funnel plot was assessed by Egger test, and* p*-value<0.05 was defined as significant publication bias. Cochrane* Q* test and* I*^*2 *^test were used to assess the heterogeneity among the selected studies. Values>50% were regarded as being indicative of moderate-to-high heterogeneity. In sensitivity analysis, we conducted ‘one-study removed' meta-analysis approach to assess the impact of a single study on the results. All statistical analyses were conducted by using Review Manager (version 5.3).

### 2.6. Role of the Funding Source

This study was supported by a National Key Basic Research Program of China (no. 2013CB531705), without any commercial entity. Our funding source had no role in the whole process of the study (study design, articles searching, data extraction, data analyses, results interpretation, and report writing). Final responsibility for the decision of publication was possessed by the corresponding author.

## 3. Results

### 3.1. Results of the Search

**Figure 1:**
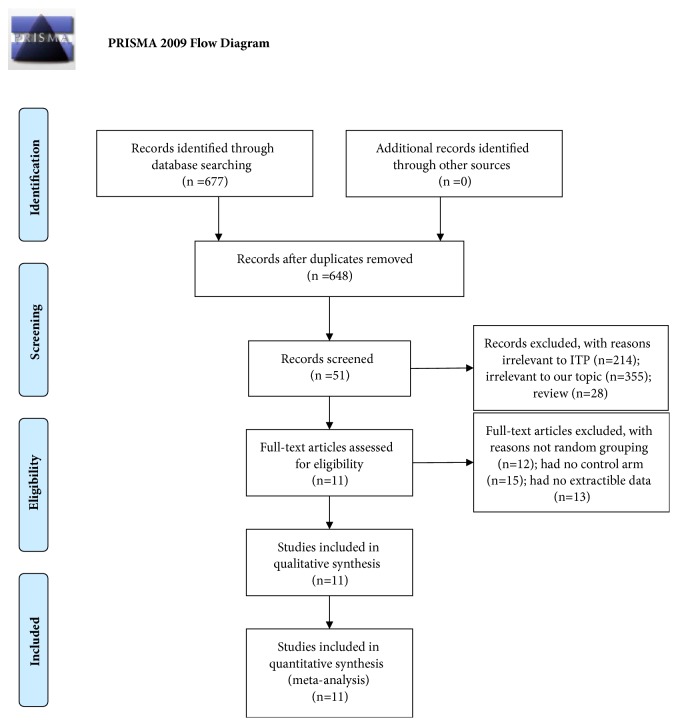
**Flow diagram of studies selection process. **ITP, primary immune thrombocytopenia.

As was presented in Figure 1, a total of 677 records were identified through electronic databases. Finally, 11 studies were included after removing the publications that duplicated or didn't satisfy the inclusion criteria. 887 cases were analyzed, with 439 in experimental arm (RTX+DXM) and 448 in control arm (DXM). Detailed study characteristics were given in Table 1 (population, interventions, and outcomes). All of the 11 trials were published between 2010 and 2017 (one was published in 2017). 11 studies were all standard randomized controlled trials, of which 3 were written in English and 8 were written in Chinese. 

Rituximab were given by the dose of 100 mg/m^2^ weekly for 4 weeks in most selected trials. There were three trials having different dose of 375 mg/m^2^ weekly for 4 weeks [18–20]. As for dexamethasone, only one trial used a different dose (12.5~25.0 mg, twice or three times a day) from that of other ten trials (40 mg daily).

Six trials compared OR, CR, and PR after 4-week intervention between the two arms. Three studies made the comparison of CR, PR, and OR at month 3. SR was compared at month 6 and month 12 respectively by three trials. Five studies reported the number of participants that relapsed during or after the intervention. Only three trials evaluated and compared the Treg cell count of participants at week 2 and week 4, respectively. As for adverse effect, infection, hyperglycemia, hypertension, and electrolyte disorder were reported most frequently in the included studies. Serious adverse effects were reported only in three trials.

### 3.2. Quality of Included Studies and Evidence

None of the 11 trials was stopped early or funded by industry. Adequate randomization was reported by all of the selected trials, with only three trials specifying the random method (S1 Figure). A total of 19 outcomes were assessed by GRADE pro software, of which 32% (6/19) was scaled as high level, 47% (9/19) was moderate level, and 21% (4/19) was low level (S2 Figure).

### 3.3. Efficacy Analysis

#### 3.3.1. Overall Response Rate

**Figure 2:**
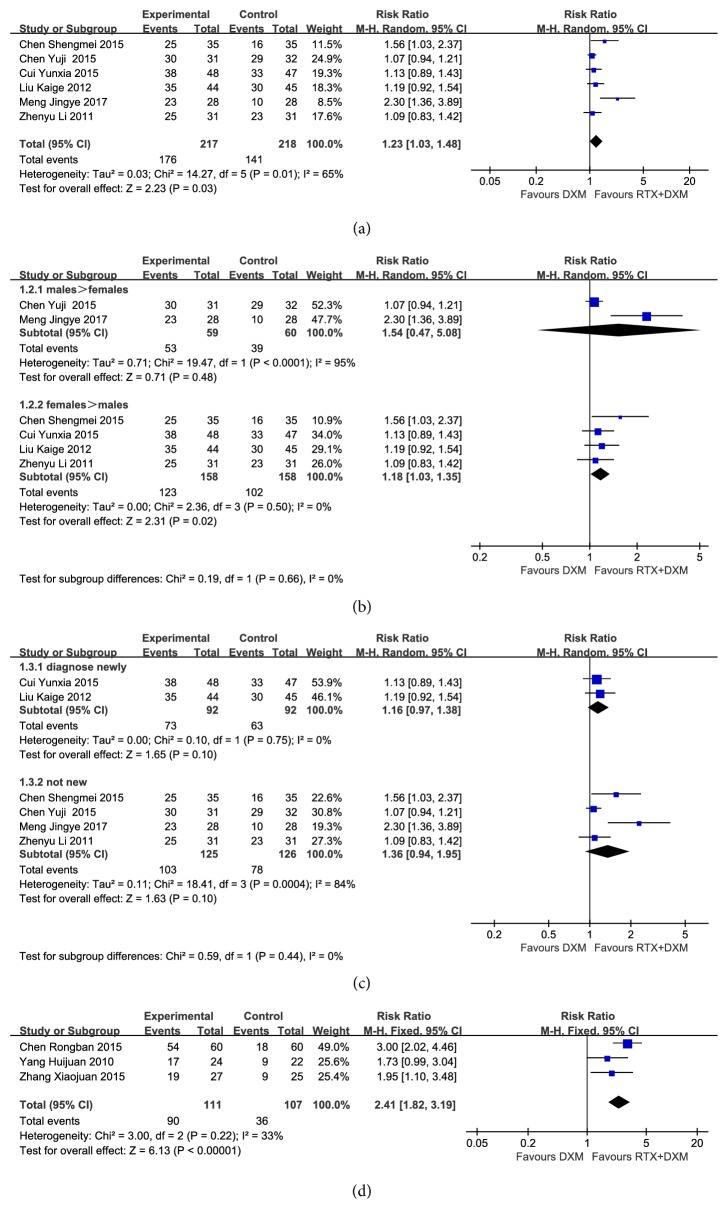
**Forest plots of relative risk in OR rate.** (a) OR rate at week 4; (b) subanalysis based on gender; (c) subanalysis based on treatment history; (d) OR rate at month 3. CI: confidence interval; M-H: Mantel-Haenszel; RR: relative risk.

The comparison of OR rate at week 4 was conducted in six trials [8, 10, 15–18] (n=435). OR rate was significantly higher in combination arm than that in monotherapy arm (RR=1.23, 95% CI:1.03-1.48, and* P*=0.03). However, high heterogeneity was found in pooled analysis (*P*=0.01, I^2^=65%) (Figure 2(a)). The gender proportion of participants varied among the six studies (Table 1), so we went further to conduct a subgroup analysis based on gender proportion (males more than females or females more than males) (Figure 2(b)). Subanalysis showed that in the first group (males more than females), heterogeneity was still high (*P*<0.05, I^2^=95%) and no significant difference was found (RR=1.54, 95%CI:0.47-5.08, and* P*=0.48). In the second group (females more than males), OR rate of combination arm was higher than that of monotherapy arm (RR=1.18, 95%CI:1.03-1.35, and* P*=0.02). No heterogeneity was observed (*P*=0.50, I^2^=0%). When we removed one study [18] that included much more males than other five studies did, the heterogeneity of total analysis decreased significantly (*P*=0.36, I^2^=9%). A subgroup analysis based on the history of treatment (newly diagnosed or not) was conducted. However, there was no significant difference between two arms in ether of the two groups (RR=1.16, 95% CI: 0.97-1.38,* P*=0.10; RR=1.36, 95% CI: 0.94-1.95, and* P*=0.10) (Figure 2(c)).

Three trials [14, 19, 20] ( n=218) reported the OR rate at month 3, pooled analysis of which showed homogeneity (*P*=0.22, I^2^=33%). OR rate at month 3 was significantly higher in combination arm than that in monotherapy arm. Fixed-effect model was applied to the analysis (RR=2.41, 95% CI: 1.82-3.19, and* P*<0.00001) (Figure 2(d)).

#### 3.3.2. Complete Response Rate

**Figure 3:**
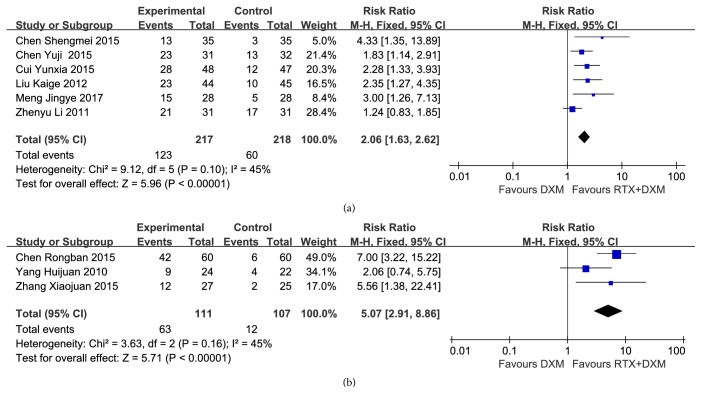
**Forest plots of relative risk in CR rate.** (a) CR rate at week 4; (b) CR rate at month 3. CI: confidence interval; M-H: Mantel-Haenszel; RR: relative risk.

Six studies [8, 10, 15–18](n=435) reported the CR rate at week 4 without significant heterogeneity (*P*=0.10, I^2^=45%). Pooled analysis by using a Fixed-effect model showed that CR rate at week 4 in combination arm was significantly higher than that in monotherapy arm (RR=2.06, 95% CI: 1.63-2.62, and* P*<0.00001) (Figure 3(a)). CR at month 3 was reported by three studies [14, 19, 20] ( n=218), and no heterogeneity was found (*P*=0.16, I^2^=45%). CR rate at month 3 in combination arm was significantly higher than that in monotherapy arm (RR=5.07, 95% CI: 2.91-8.86, and* P*<0.00001) (Figure 3(b)).

#### 3.3.3. Partial Response Rate

**Figure 4:**
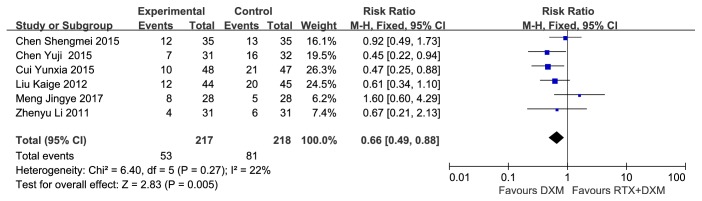
**Forest plots of relative risk in PR rate at week 4.** CI: confidence interval; M-H: Mantel-Haenszel; RR: relative risk.

PR rate at week 4 was reported by six studies [8, 10, 15–18](n=435), pooled analysis of which turned out homogenous (*P*=0.27, I^2^=22%). Analysis conducted by a Fixed-effect model showed that PR rate at week 4 in monotherapy arm was significantly higher than that in combination arm (RR=0.66, 95% CI: 0.49-0.88,* P*=0.005) (Figure 4). Three trials [14, 19, 20](n=218) compared the PR rate at month 3, and no heterogeneity was found (*P*=0.76, I^2^=0%). However, the analysis result showed no significant difference between two arms (RR=1.08, 95% CI: 0.67-1.74,* P*=0.76).

#### 3.3.4. Sustained Response Rate

**Figure 5:**
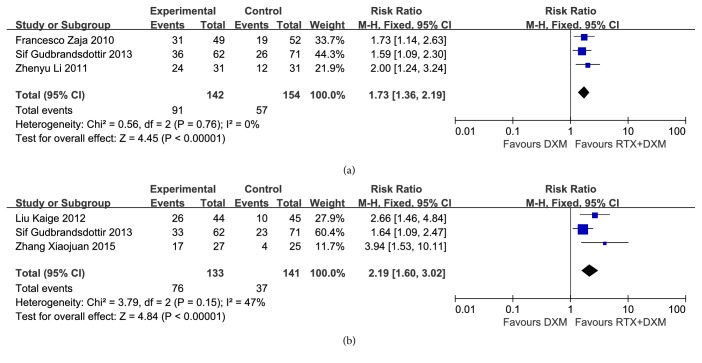
**Forest plots of relative risk in SR rate.** (a) SR at month 6; (b) SR at month 12. CI: confidence interval; M-H: Mantel-Haenszel; RR: relative risk.

SR rate at month 6 [8, 11, 12] (n=296) and month 12 [11, 17, 19] (n=274) was reported by three studies, respectively, both of which showed no significant heterogeneity (*P*=0.76, I^2^=0%;* P*=0.15, I^2^=47%). Consequently, two analyses were both conducted by a Fixed-effect model and showed that SR rate was significantly higher in combination arm than that in monotherapy arm both at month 6 and month 12 (RR=1.73, 95% CI: 1.36-2.91, and* P*<0.00001; RR=2.19, 95% CI: 1.60-3.02, and* P*<0.00001) (Figure 5).

### 3.4. Treg Cell Count

**Figure 6:**
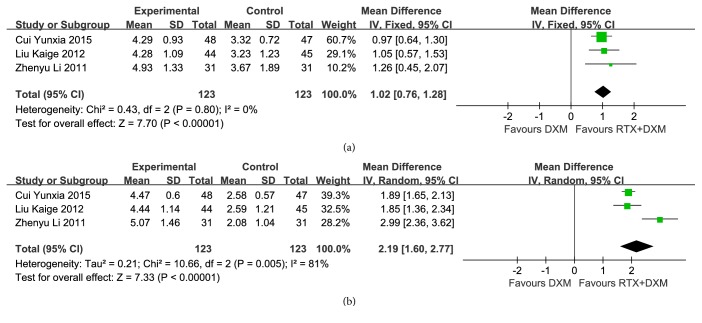
**Forest plots of MD in Treg cell count.** (a) Treg cell count at week 2; (b) Treg cell count at week 4. CI: confidence interval; IV: Inverse Variance; MD: Mean Difference.

Only three studies [8, 10, 17] (n=246) evaluated the Treg cell count of participants and reported Treg cell count at baseline, week 2 and week 4, respectively. There was no statistical difference at baseline between two arms (*P*=0.97), meaning that the comparison was feasible. Pooled analysis of Treg cell count at week 2 indicated that combination arm had a better effect compared to monotherapy arm (MD=1.02, 95% CI: 0.76-1.28, and* P*<0.00001). Besides, no heterogeneity was found (*P*=0.80, I^2^=0%) (Figure 6(a)). Analysis of Treg cell count at week 4 still showed a better effect of experimental arm to control arm (MD=2.19, 95% CI: 1.60-2.77, and* P*<0.00001). However, high heterogeneity was observed (*P*=0.005, I^2^=81%) (Figure 6(b)). When we removed one study which recruited much younger participants than other studies did [8], the heterogeneity disappeared (*P*=0.88, I^2^=0%).

#### 3.4.1. Relapse Rate

Five trials [8, 10–12, 20] (n=437) reported the relapse rate without heterogeneity (*P*=0.21, I^2^=32%), and no significant difference was found in the pooled analysis (RR=0.63, 95% CI: 0.40-1.02, and* P*=0.06). A subgroup analysis was conducted based on the duration of follow-up (shorter than 12 months or 12 months and longer). However, there was still no significant difference in either of the two subgroups (*P*=0.57;* P*=0.07) (Figure S3).

### 3.5. Safety Profile

Only three trials [11, 12, 19] (n=286) reported serious AE. Through a Fixed-effect method, no heterogeneity was observed (*P*=0.67, I^2^=0%), and no significant difference was found either (RR=1.93, 95% CI: 1.00-3.71, and* P*=0.05) (Figure S4).

The results of pooled analyses for other adverse events was as follows: infection [12, 14–19] (n=684) (RR=1.19, 95% CI: 0.86-1.65, and* P*=0.28); hyperglycemia[8, 11, 14–16, 19, 20] (n=546) (RR=0.90, 95% CI: 0.58-1.38, and* P*=0.63); hypertension [8, 14, 16, 19] (n=367) (RR=1.19, 95% CI: 0.75-1.89, and* P*=0.45); electrolyte disorder [14–17, 19] (n=394) (RR=1.13, 95% CI: 0.83-1.54, and* P*=0.42); fever [11, 12, 18] (n=290) (RR=4.30, 95% CI: 0.92-20.06, and* P*=0.06); rash [12, 17, 18] (n=246) (RR=2.05, 95% CI: 0.53-7.98, and* P*=0.30). No heterogeneous was found in pooled analyses of all of the adverse events above (*P*>0.1).

### 3.6. Publication Bias

**Figure 7:**
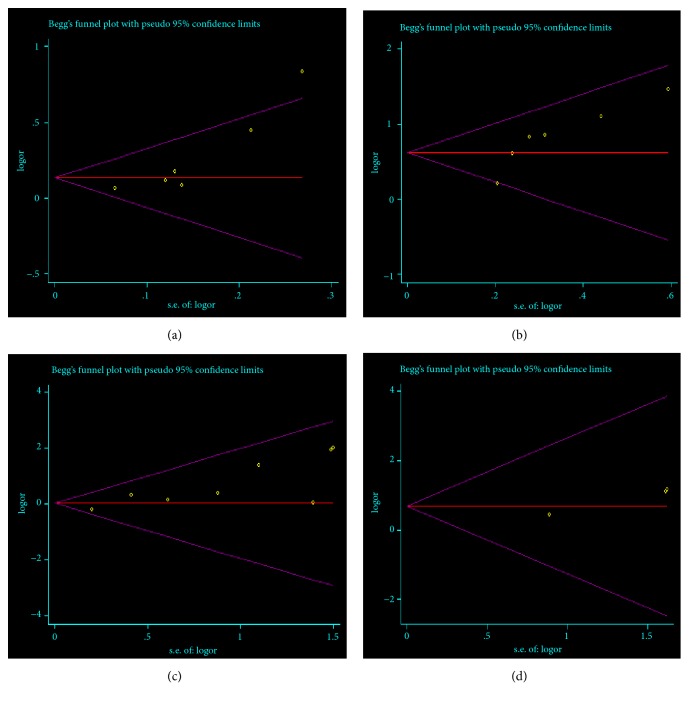
**Funnel plots of publication bias. **(a) publication bias of OR rate at week 4; (b) publication bias of CR rate at week 4; (c) publication bias of incidence in infection; (d) publication bias of incidence in rash.

Egger's test showed that there was a significant publication bias of studies on OR rate at week 4 (t=3.50,* P*=0.025), CR rate at week 4 (t=3.94,* P*=0.017), incidence of infection (t=4.09,* P*=0.006), and rash (t=22.41,* P*=0.028). The funnel plots of publication bias were shown in Figure 7.

## 4. Discussion

To our knowledge, this is the first meta-analysis assessing the efficacy and safety of the combination treatment of rituximab and dexamethasone for adults with ITP. Our results showed that compared to dexamethasone monotherapy, combination treatment with rituximab can improve the long-term sustained response rate, with no increase in serious AEs, infection, hyperglycemia, hypertension, electrolyte disorder, fever, and rash. Moreover, combination treatment showed a far better efficacy of Treg cell upregulating early in the observation. However, dexamethasone combining with rituximab still could not reduce the incidence of relapse in adults with ITP.

The pathophysiology of ITP is very complicated, which involves pathologic autoantibodies, destruction of platelets mediated by CD8^+^ cytotoxic T-cells, imbalance in T-cell cytokines, T-cell subsets, and impaired megakaryocyte maturation, with each pathologic mechanism playing different parts in different patients [21]. Moreover, relevant factors such as complications of specific therapy, tolerance of side effects, age, and lifestyle may contribute to the determination of regimen to different extent [4]. Therefore, the investigation and management of ITP patients vary quite widely.

Corticosteroids are standard initial treatments with a rapid efficacy. Dexamethasone, for example, has been reported to provide an initial response after several days to several weeks [22, 23]. However, corticosteroid-related complications have limited its long-term utility and efficacy. Consequently, more and more studies about combination regimens with second-line agents are conducted to improve the efficacy and obtain long-term remission [24–27]. Rituximab can induce a potent and prolonged B cell depletion that can last several months impairing the antibodies production, providing long-term response rates of 40% and 33% at 1 and 2 years of follow-up, respectively [28, 29]. Patients that relapse early or are resistant to the treatment can particularly benefit from RTX application, which therefore is a promising candidate to a second line approach such as splenectomy. However, most of the studies on combination regimen are reviews and single-arm trials. There are still so few RCTs that it is necessary to conduct a meta-analysis to collect and synthesize the data of each study, assessing the efficacy and safety of the combination treatment of dexamethasone and rituximab.

In our report, the combination arm (RTX +DXM) obtained a OR rate of 81% at week 4, while the monotherapy (DXM) arm obtained 65% (*P*=0.03). Given a high heterogeneity, a subgroup analysis based on gender proportion (males more than females or females more than males) was conducted, which showed that this trend persisted only in the second group (females more than males), and the heterogeneity decreased significantly (*P*=0.50, I^2^=0%). It is indicated that females might have a better OR rate at 4 weeks than males do (*P*=0.02). Similar subgroup analysis could not be done in OR rate at 3 months to confirm this gender-relevant trend, because only 3 trials reported OR rate at 3 months. Since the history of treatment of participants varied greatly among the six studies, a subgroup analysis based on the history of treatment (newly diagnosed or not) was conducted, which turned out no significant difference between two arms in ether of the two groups. Besides, publication bias was found in the pooled analysis of OR at week 4, which made the results instable. Comparison at month 3 also showed a better OR rate in combination arm (81%) than that in monotherapy arm (34%) (*P*<0.01). This result was quite different from the international consensus report published in 2010, which suggested that dexamethasone and rituximab could provide an approximate initially response rate of 90% and 60%, respectively [4]. It might be because all of the three trials [14, 19, 20] chose patients with chronic ITP, persistent ITP, and refractory ITP, respectively, as participants.

CR rate at week 4 was 57% in combination arm and 28% in monotherapy arm, respectively, (*P*<0.01), which was consistent with CR rate at month 3 (57% vs 11%) (*P*<0.01). The definition of PR varied among the included studies, most of which was≥30×10^9^/L. Only two trials [8, 20] defined PR by a platelet count≥50×10^9^/L, which reported PR rate at week 4 and month 3, respectively. Our result showed that PR rate at week 4 in monotherapy arm was higher than that of combination arm (37% vs 24%) (*P*=0.005). When we removed a single study with a different definition of PR [8], the trend still persisted (*P*=0.006). Considering that OR rate and CR rate at week 4 were both higher in combination arm and OR rate means CR rate plus PR rate, this result indicated that participants obtaining PR was much more than that obtaining CR in monotherapy arm.

SR rate at month 6 and month 12 were available in only three studies, respectively. However, a total of 296 and 274 participants were involved in the pooled analyses. Combination arm showed a SR rate of 64% after 6 months, which was higher than that of monotherapy arm (37%) (*P*<0.01). Similarly, SR rate at month 12 in combination arm was also better than that in monotherapy arm (57% vs 26%) (*P*<0.01). In short, dexamethasone combined with rituximab can provide a much better long-term efficacy than dexamethasone alone does. Our results were consistent with the literature, which was reported previously that rituximab could provide a long-term remission lasting up to 5 years in 15%~20% of initially treated patients [30]. A prospective, open-label, single-arm phase 2 trial showed that 40% of the participants accepting rituximab had a platelet count of 30×10^9^/L after two years of the follow-up [28].

Patients with ITP have a lack of peripheral tolerance, which can be mediated by T regulatory lymphocytes (Treg cells) [31, 32]. It has been reported that clonal Th cells that fail to be suppressed by Treg cells can drive the production of autoantibody in ITP [26]. ITP patients have a defective Treg cells compartment that can be modulated by a B cell-targeted therapy [9, 33]. Actually, the regulation effect in T-cell cytokines and T-cell subsets by rituximab has been reported by studies of many other autoimmune disorders [34–36]. In our report, Treg cell counts were available in three trials, which were evaluated on baseline, week 2 and week 4, respectively. Pooled analyses showed that there was no statistical difference at baseline between two arms (*P*=0.97), whereas, Treg cell count was higher in combination arm than that in monotherapy arm both at week 2 (*P*<0.01) and week 4 (*P*<0.01). However, high heterogeneity was observed in the comparison at week 4. When we removed one study [8], the heterogeneity disappeared (*P*=0.88, I^2^=0%). We noticed that participants in the removed study (median age: 25 years) were much younger than that in the other two studies (median age: 42 years; 40 years). Unfortunately, subgroup analysis based on age could not be conducted because too few studies were included.

In our report, combination arm had a relapse rate of 17%, and monotherapy arm was 27%. However, the pooled analysis showed no significant difference between two arms (*P*=0.06). We went further with a subgroup analysis based on the duration of follow-up (<12 months or ≥12 months), which eventually still showed no significant difference in either of the two subgroups (*P*=0.57;* P*=0.07). Our results indicated that RTX combining DXM could not reduce the incidence of relapse in adults with ITP.

As for safety, the main goal of this meta-analysis was to confirm whether the combination treatment of RTX and DXM would increase the incidence of adverse effects that the DXM monotherapy had. In our study, only three trials reported serious AE without a significant difference between two arms by pooled analysis (*P*=0.05). A total of 4 participants (80 to 84 years old) deceased from what were not considered to be treatment-related by the investigator [11]. Because of the depletion of B-lymphocytes, infection is a major-concerned AE both for dexamethasone and rituximab. Our results showed no significant difference between combination arm and monotherapy arm (*P*=0.28). The pooled analyses of other AEs (hyperglycemia, hypertension, electrolyte disorder, fever, and rash) also found no significant difference between two arms, which meant the combination treatment of RTX and DXM was quite safe in the treatment with ITP.

Although 11 studies included were all standard RCTs with 79% moderate-to-high level evidences (GRADE pro scale), there were still several limitations in this meta-analysis. First, the observation points of each study varied, and pooled analyses had to be done discretely, which caused fewer data-collection in the analysis of each outcome. Second, the dose of rituximab in three trials were standard dose (375mg/m^2^), whereas other eight trials used low-dose rituximab (100mg). However, it was difficult for us to conduct subanalyses based on dose because not every outcome was reported by all of the three trials (375mg/m^2^). The significant heterogeneity in the analyses of two outcomes (OR rate at week 4 and Treg cell count at week 4) should be the third limitation. In addition, the history of treatment of participants varied greatly among the studies. We did a subgroup analysis in OR rate at week 4 based on history of treatment (newly diagnosed or not). However, too few studies were included to conduct further analysis in other outcomes, which made it difficult to assess the efficacy of combination treatment in different phase of ITP. Lastly, given that ITP is a quite heterogenous disease, some relapses may happen even after several months from the diagnosis, which makes long-term follow-up necessary for relapse rate assessment. However, only three trials did an adequate follow-up (≥12 months) in our report, which might contribute to an underestimated relapse rate for both combination treatment arm and monotherapy arm.

Despite that more studies are needed to clarify the optimal approach to the application of this combination treatment (dose and timing), this meta-analysis clearly confirms that rituximab combined with dexamethasone can provide a better long-term response in the treatment of adults with ITP and will not increase the risk of adverse effects.

## Figures and Tables

**Table 1:** Study characteristics.

Study	Median age (year)		Gender (M/F)		Follow-up	Dose of RTX	Dose of DXM	Outcomes
	RTX+DXM	DXM	RTX+DXM	DXM				
Chen Rongban et al. (2015)	46.0	48.0	33/27	32/28	4 weeks	100mg, weekly, 28 d	40mg, qd, 1~4d/cycle×2~4	CR, PR, OR on month 3; AE
Chen Shengmei et al. (2015)	46.7	46.9	13/22	15/20	4 weeks	100mg, weekly, 28 d	40mg, qd, 1~4d	CR, PR, OR on week 4; AE
Chen Yuji et al. (2015)	39.9	39.9	16/15	16/16	4 weeks	100mg, weekly, 28 d	40mg, qd, 1~4d	CR, PR, OR on week 4; AE
Cui Yunxia et al. (2015)	41.0	42.0	21/27	19/28	6 months	100mg, weekly, 28 d	12.5~25mg, bid~qid, 1~4d	CR, PR, OR on week 4; R; Treg cell count
Francesco Zaja et al. (2010)	49.0	47.0	22/27	19/33	6 months	375mg/m^2^, weekly, 28d	40mg, qd, 1~4d	SR at month 12; R; AE; serious AE
Liu Kaige et al. (2012)	40.0	40.0	17/24	18/27	12 months	100mg, weekly, 28 d	40mg, qd, 1~4d	CR, PR, OR on week 4; SR at month 12; Treg cell count; AE
Meng Jingye et al. (2017)	38.0	35	18/10	20/8	4 weeks	100mg, weekly, 28 d	40mg, qd, 1~4d	CR, PR, OR on week 4; AE
Sif Gudbrandsdottir et al. (2013)	51.0	58.0	26/36	37/34	24 months	375mg/m^2^, weekly, 28d	40mg, qd, 1~4d	SR at month 6 and 12; R; AE; serious AE
Yang Huijuan et al. (2010)	50.2	47.3	10/14	9/13	3 months	375mg/m^2^ weekly, 28d	40mg, qd, 1~4d/cycle×4	CR, PR, OR on month 3; R; AE
Zhang Xiaojuan et al. (2015)	38.0		20/32		4 weeks	100mg, weekly, 28 d	40mg, qd, 1~4d	CR, PR, OR on month 3; SR at month 12; AE; serious AE
Zhenyu Li et al. (2011)	26.0	24.0	13/18	12/19	12 months	100mg, weekly, 28 d	40mg, qd, 1~4d	CR, PR, OR on week 4; SR at month 6; R; Treg cell count; AE

RTX: rituximab; DXM: dexamethasone; M: male; F: female; qid: once a day; bid: twice a day; qid four times a day; CR: complete response (platelet count*⩾*100×10^9^/L); PR: partial response (platelet count*⩾*30×10^9^/L); OR: overall response (OR=CR+PR); SR: sustained response (platelet count*⩾*50×10^9^/L at month 6); R: relapse (dropping in platelet count to⩽30×10^9^/L following a CR or PR); AE: adverse effect.

## Supplementary Materials

Supplementary 1 S1 Figure. Risk of bias summary and risk of bias graph according to Cochrane Risk of Bias assessment tool.

Supplementary 2 S2 Figure. Assessment of evidences by GRADE pro software.

Supplementary 3 S3 Figure. Forest plots of relative risk in relapse rate.

Supplementary 4 S4 Figure. Forest plots of relative risk in serious adverse effects.

Supplementary 5 S5 Figure. PRISMA 2009 Checklist.

## References

[1] Cines D. B., Bussel J. B., Liebman H. A., Luning Prak E. T. (2009). The ITP syndrome: pathogenic and clinical diversity. *Blood*.

[2] Chong B. H., Lee J.-W., Jang J. H. (2017). International ITP Registry with Focus on the Asia Pacific Region: Update on Preliminary Findings of Epidemiological and Clinical Data. *Blood*.

[3] Chong B. H., Lee J.-W., Jang J. H. (2017). ITP Patients in the Asia Pacific: Are They Different?. *Blood*.

[4] Provan D., Stasi R., Newland A. C. (2010). International consensus report on the investigation and management of primary immune thrombocytopenia. *Blood*.

[5] Patel V. L., Mahévas M., Lee S. Y. (2012). Outcomes 5 years after response to rituximab therapy in children and adults with immune thrombocytopenia. *Blood*.

[6] Khellaf M., Charles-Nelson A., Fain O. (2014). Safety and efficacy of rituximab in adult immune thrombocytopenia: Results from a prospective registry including 248 patients. *Blood*.

[7] Al-Habsi K., Al-Khabori M., Al-Muslahi M. (2015). Rituximab leads to long remissions in patients with chronic immune thrombocytopenia. *Oman Medical Journal*.

[8] Li Z., Mou W., Lu G. (2011). Low-dose rituximab combined with short-term glucocorticoids up-regulates Treg cell levels in patients with immune thrombocytopenia. *International Journal of Hematology*.

[9] Stasi R., Cooper N., Poeta G. D. (2008). Analysis of regulatory T-cell changes in patients with idiopathic thrombocytopenic purpura receiving B cell depleting therapy with rituximab. *Blood*.

[10] Cui Y. X. (2015). Effect of large dose of dexamethasone combined with rituximab on CD^4+^CD^5+^ Treg cells in patients with primary immune thrombocytopenia. *Chinese Journal of New Clinical Medicine*.

[11] Gudbrandsdottir S., Birgens H. S., Frederiksen H. (2013). Rituximab and dexamethasone vs dexamethasone monotherapy in newly diagnosed patients with primary immune thrombocytopenia. *Blood*.

[12] Zaja F., Baccarani M., Mazza P. (2010). Dexamethasone plus rituximab yields higher sustained response rates than dexamethasone monotherapy in adults with primary immune thrombocytopenia. *Blood*.

[13] Neunert C., Lim W., Crowther M., Cohen A., Solberg L., Crowther M. A. (2011). The American Society of Hematology 2011 evidence-based practice guideline for immune thrombocytopenia. *Blood*.

[14] Chen R. B. (2015). Clinical effect observation of glucocorticoid in combination with small dose of rituxan in treating primary immune thrombocytopenia. *Clinical Medicine*.

[15] Chen S. M., Zhou D.-B. (2015). Clinical observation of rituximab treatment of chronic primary immune thrombocytopenia in adults. *Journal of China Prescription Drug*.

[16] Chen Y. (2015). Observation the Effect of Low-dose Rituximab Combined with Dexamethasone Treating Adult Persistent Immune Thrombocytopenia (ITP). *China Continuing Medical Education*.

[17] Kaige L. (2012). *Conventional dose prednisone, high dose dexamethasone and high dose dexamethasone combined with low dose rituximab therapy for newly diagnosis immune thrombocytopenia. MM.Sc. Thesis*.

[18] Jingye M., Chan L., Ziyi L. (2017). Clinical study of glucocorticoids combined with low-dose of rituximab in the treatment of refractory primary immune thrombocytopenia. *Clinical Medicine*.

[19] Xiaojuan Z., Shuxia G., Ronghua C. (2015). Observation of low-dose rituximab combined with dexamethasone in the treatment of adult persistent primary immune thrombocytopenia. *Shandong Medical Journal*.

[20] Huijuan Y. (2010). Observation of rituximab combined with dexamethasone in the treatment of refractory primary immune thrombocytopenia. *Aerospace Medicine*.

[21] Lambert M. P., Gernsheimer T. B. (2017). Clinical updates in adult immune thrombocytopenia. *Blood*.

[22] Stasi R., Stipa E., Masi M. (1995). Long-Term observation of 208 adults with chronic idiopathic thrombocytopenic purpura. *American Journal of Medicine*.

[23] Ben-Yehuda D., Gillis S., Eldor A. (1994). Clinical and therapeutic experience in 712 israeli patients with idiopathic thrombocytopenic purpura. Israeli ITP Study Group. *Acta Haematologica*.

[24] Li Y., Huang Q., Wang C., Muhebaier, An L., Wang X. (2016). Efficacy and safety of high-dose dexamethasone combined with rhTPO for newly diagnosed adults with severe immune thrombocytopenia. *Zhonghua Xue Ye Xue Za Zhi*.

[25] Han X. D., Zhou J., Yu F. K. (2016). Rituximab and Dexamethasone Combined with Cyclophosphamide for Treatment of Relapsed and Refractory Immune Thrombocytopenia. *Zhongguo Shi Yan Xue ye Xue Za Zhi*.

[26] Gómez-Almaguer D., Herrera-Rojas M. A., Jaime-Pérez J. C. (2014). Eltrombopag and high-dose dexamethasone as frontline treatment of newly diagnosed immune thrombocytopenia in adults. *Blood*.

[27] Bussel J. B., Lee C. S., Seery C. (2014). Rituximab and three dexamethasone cycles provide responses similar to splenectomy in women and those with immune thrombocytopenia of less than two years duration. *Haematologica*.

[28] Godeau B., Porcher R., Fain O. (2008). Rituximab efficacy and safety in adult splenectomy candidates with chronic immune thrombocytopenic purpura: Results of a prospective multicenter phase 2 study. *Blood*.

[29] Patel V. L., Mahévas M., Stasi R. (2010). Long-term outcome following B-cell depletion therapy with rituximab in children and adults with immune thrombocytopenia. *Blood*.

[30] Arnold D. M., Dentali F., Crowther M. A. (2007). Systematic review: Efficacy and safety of rituximab for adults with idiopathic thrombocytopenic purpura. *Annals of Internal Medicine*.

[31] Yu J., Heck S., Patel V. (2008). Defective circulating CD25 regulatory T cells in patients with chronic immune thrombocytopenic purpura. *Blood*.

[32] Sakaguchi S. (2005). Naturally arising Foxp3-expressing CD25^+^CD4^+^ regulatory T cells in immunological tolerance to self and non-self. *Nature Immunology*.

[33] Stasi R., del Poeta G., Stipa E. (2007). Response to B-cell-depleting therapy with rituximab reverts the abnormalities of T-cell subsets in patients with idiopathic thrombocytopenic purpura. *Blood*.

[34] Sfikakis P. P., Souliotis V. L., Fragiadaki K. G., Moutsopoulos H. M., Boletis J. N., Theofilopoulos A. N. (2007). Increased expression of the FoxP3 functional marker of regulatory T cells following B cell depletion with rituximab in patients with lupus nephritis. *Clinical Immunology*.

[35] Bhattacharjee R., De D., Handa S., Minz R. W., Saikia B., Joshi N. (2017). Assessment of the Effects of Rituximab Monotherapy on Different Subsets of Circulating T-Regulatory Cells and Clinical Disease Severity in Severe Pemphigus Vulgaris. *Dermatology*.

[36] Roccatello D., Sciascia S., Di Simone D. (2016). New insights into immune mechanisms underlying response to Rituximab in patients with membranous nephropathy: a prospective study and a review of the literature. *Autoimmunity Reviews*.

