# Supplementary material for: Efficacy and Safety of the Combination Treatment of Rituximab and Dexamethasone for Adults with Primary Immune Thrombocytopenia (ITP): A Meta-Analysis
Source: Biomed Res Int. 2018 Dec 12;2018:1316096. doi: 10.1155/2018/1316096 (PMC6311778; doi:10.1155/2018/1316096)
Supplement: Supplementary 1 — S1 Figure. Risk of bias summary and risk of bias graph according to Cochrane Risk of Bias assessment tool. [file 1316096.f1.pdf]

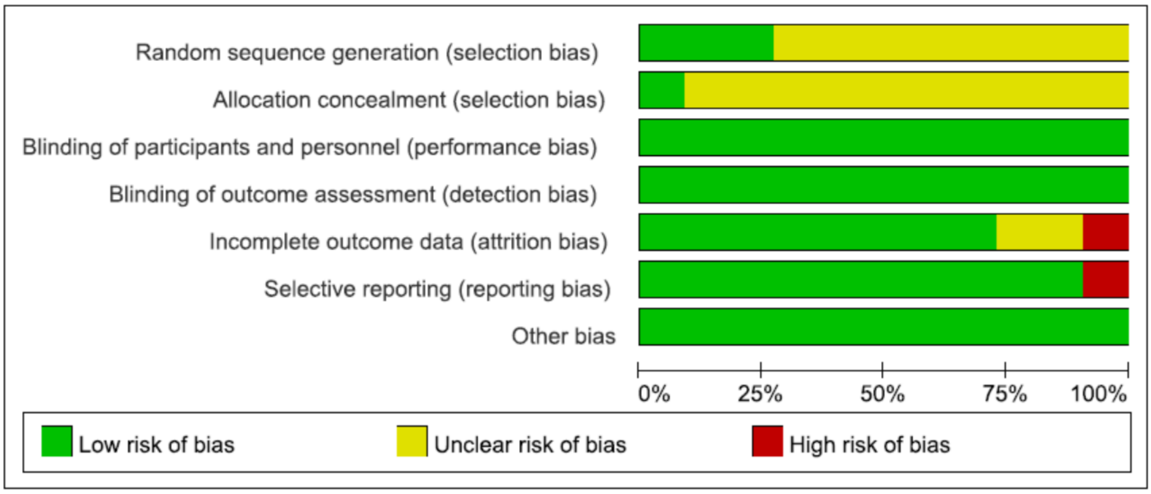

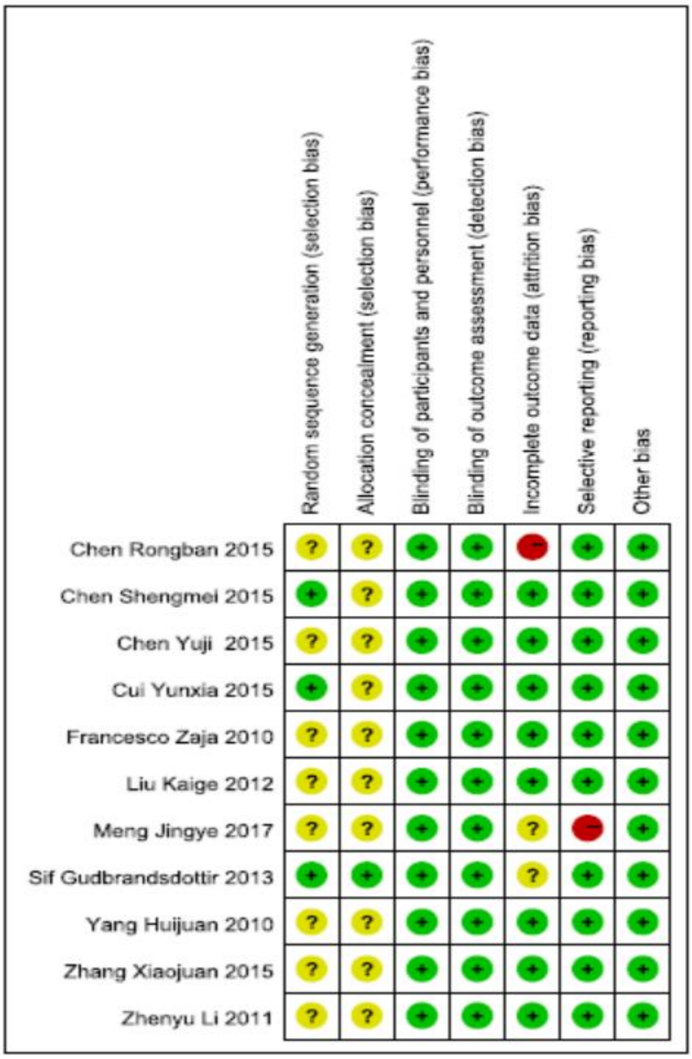

S1_Fig. Risk of bias summary and risk of bias graph according to Cochrane Risk of Bias assessment tool.
