# Supplementary material for: Efficacy and Safety of the Combination Treatment of Rituximab and Dexamethasone for Adults with Primary Immune Thrombocytopenia (ITP): A Meta-Analysis
Source: Biomed Res Int. 2018 Dec 12;2018:1316096. doi: 10.1155/2018/1316096 (PMC6311778; doi:10.1155/2018/1316096)
Supplement: Supplementary 2 — S2 Figure. Assessment of evidences by GRADE pro software. [file 1316096.f2.pdf]

OR in 28 days for primary immune thrombocytopenia

Patient or population: patients with primary immune thrombocytopenia
Settings:
Intervention: OR in 28 days

Outcomes	Illustrative comparative risks* (95% CI)		Relative effect (95% CI)	No of Participants (studies)	Quality of the evidence (GRADE)	Comments
	Assumed risk<br>**Control**	Corresponding risk<br>**OR in 28 days**				
**OR in 28 days**	**Study population**		**RR 1.26**<br>(1.12 to 1.41)	435<br>(6 studies)	⊕⊕⊖⊖<br>**low**[1,2]	
	**647 per 1000**	**815 per 1000**<br>(725 to 912)				
	**Medium risk population**					
	**684 per 1000**	**862 per 1000**<br>(766 to 964)				

*The basis for the **assumed risk** (e.g. the median control group risk across studies) is provided in footnotes. The **corresponding risk** (and its 95% confidence interval) is based on the assumed risk in the comparison group and the **relative effect** of the intervention (and its 95% CI).

CI: Confidence interval; **RR:** Risk ratio;

GRADE Working Group grades of evidence
High quality: Further research is very unlikely to change our confidence in the estimate of effect.
Moderate quality: Further research is likely to have an important impact on our confidence in the estimate of effect and may change the estimate.
Low quality: Further research is very likely to have an important impact on our confidence in the estimate of effect and is likely to change the estimate.
Very low quality: We are very uncertain about the estimate.

[1] Allocation concealment is uncertain
[2] Funnel plot is not symmetric.

OR in 3 months for primary immune thrombocytopenia

Patient or population: patients with primary immune thrombocytopenia
Settings:
Intervention: OR in 3 months

Outcomes	Illustrative comparative risks* (95% CI)		Relative effect (95% CI)	No of Participants (studies)	Quality of the evidence (GRADE)	Comments
	Assumed risk<br>**Control**	Corresponding risk<br>**OR in 3 months**				
**OR in 3 months**	**Study population**		**RR 2.41**<br>(1.82 to 3.19)	218<br>(3 studies)	⊕⊕⊕⊕<br>**high**[1]	
	**336 per 1000**	**810 per 1000**<br>(612 to 1000)				
	**Medium risk population**					
	**360 per 1000**	**868 per 1000**<br>(655 to 1000)				

*The basis for the **assumed risk** (e.g. the median control group risk across studies) is provided in footnotes. The **corresponding risk** (and its 95% confidence interval) is based on the assumed risk in the comparison group and the **relative effect** of the intervention (and its 95% CI).

CI: Confidence interval; **RR:** Risk ratio;

GRADE Working Group grades of evidence
High quality: Further research is very unlikely to change our confidence in the estimate of effect.
Moderate quality: Further research is likely to have an important impact on our confidence in the estimate of effect and may change the estimate.
Low quality: Further research is very likely to have an important impact on our confidence in the estimate of effect and is likely to change the estimate.
Very low quality: We are very uncertain about the estimate.

[1] Randomized method was not mentioned.

CR in 28 days for primary immune thrombocytopenia

Patient or population: patients with primary immune thrombocytopenia
Settings:
Intervention: CR in 28 days

Outcomes	Illustrative comparative risks* (95% CI)		Relative effect (95% CI)	No of Participants (studies)	Quality of the evidence (GRADE)	Comments
	Assumed risk<br>**Control**	Corresponding risk<br>**CR in 28 days**				
**CR in 28 days**	**Study population**		**RR 2.06**<br>(1.63 to 2.62)	435<br>(6 studies)	⊕⊕⊖⊖<br>**low**[1,2]	
	**275 per 1000**	**566 per 1000**<br>(448 to 720)				
	**Medium risk population**					
	**239 per 1000**	**492 per 1000**<br>(390 to 626)				

*The basis for the **assumed risk** (e.g. the median control group risk across studies) is provided in footnotes. The **corresponding risk** (and its 95% confidence interval) is based on the assumed risk in the comparison group and the **relative effect** of the intervention (and its 95% CI).

CI: Confidence interval; **RR:** Risk ratio;

GRADE Working Group grades of evidence
High quality: Further research is very unlikely to change our confidence in the estimate of effect.
Moderate quality: Further research is likely to have an important impact on our confidence in the estimate of effect and may change the estimate.
Low quality: Further research is very likely to have an important impact on our confidence in the estimate of effect and is likely to change the estimate.
Very low quality: We are very uncertain about the estimate.

[1] Concealment of allocation was uncertain
[2] There was a publication bias.

CR in 3 months for primary immune thrombocytopenia

Patient or population: patients with primary immune thrombocytopenia
Settings:
Intervention: CR in 3 months

Outcomes	Illustrative comparative risks* (95% CI)		Relative effect (95% CI)	No of Participants (studies)	Quality of the evidence (GRADE)	Comments
	Assumed risk **Control**	Corresponding risk **CR in 3 months**				
**CR in 3 months**	**Study population**		**RR 5.07** (2.91 to 8.86)	218 (3 studies)	⊕⊕⊕⊕ **high**	
	**112 per 1000**	**568 per 1000** (326 to 992)				
	**Medium risk population**					
	**100 per 1000**	**507 per 1000** (291 to 886)				

*The basis for the **assumed risk** (e.g. the median control group risk across studies) is provided in footnotes. The **corresponding risk** (and its 95% confidence interval) is based on the assumed risk in the comparison group and the **relative effect** of the intervention (and its 95% CI).

CI: Confidence interval; **RR:** Risk ratio;

GRADE Working Group grades of evidence
High quality: Further research is very unlikely to change our confidence in the estimate of effect.
Moderate quality: Further research is likely to have an important impact on our confidence in the estimate of effect and may change the estimate.
Low quality: Further research is very likely to have an important impact on our confidence in the estimate of effect and is likely to change the estimate.
Very low quality: We are very uncertain about the estimate.

PR in 28 days for primary immune thrombocytopenia

Patient or population: patients with primary immune thrombocytopenia
Settings:
Intervention: PR in 28 days

Outcomes	Illustrative comparative risks* (95% CI)		Relative effect (95% CI)	No of Participants (studies)	Quality of the evidence (GRADE)	Comments
	Assumed risk **Control**	Corresponding risk **PR in 28 days**				
**PR in 28 days**	**Study population**		**RR 0.66** (0.49 to 0.88)	435 (6 studies)	⊕⊕⊕⊖ **moderate**[1]	
	**372 per 1000**	**246 per 1000** (182 to 327)				
	**Medium risk population**					
	**408 per 1000**	**269 per 1000** (200 to 359)				

*The basis for the **assumed risk** (e.g. the median control group risk across studies) is provided in footnotes. The **corresponding risk** (and its 95% confidence interval) is based on the assumed risk in the comparison group and the **relative effect** of the intervention (and its 95% CI).

CI: Confidence interval; **RR:** Risk ratio;

GRADE Working Group grades of evidence
High quality: Further research is very unlikely to change our confidence in the estimate of effect.
Moderate quality: Further research is likely to have an important impact on our confidence in the estimate of effect and may change the estimate.
Low quality: Further research is very likely to have an important impact on our confidence in the estimate of effect and is likely to change the estimate.
Very low quality: We are very uncertain about the estimate.

[1] Concealment of allocation was uncertain.

PR in 3 months for primary immune thrombocytopenia

Patient or population: patients with primary immune thrombocytopenia
Settings:
Intervention: PR in 3 months

Outcomes	Illustrative comparative risks* (95% CI)		Relative effect (95% CI)	No of Participants (studies)	Quality of the evidence (GRADE)	Comments
	Assumed risk **Control**	Corresponding risk **PR in 3 months**				
**PR in 3 months**	**Study population**		**RR 1.08** (0.67 to 1.74)	218 (3 studies)	⊕⊕⊕⊕ **high**	
	**224 per 1000**	**242 per 1000** (150 to 390)				
	**Medium risk population**					
	**227 per 1000**	**245 per 1000** (152 to 395)				

*The basis for the **assumed risk** (e.g. the median control group risk across studies) is provided in footnotes. The **corresponding risk** (and its 95% confidence interval) is based on the assumed risk in the comparison group and the **relative effect** of the intervention (and its 95% CI).

CI: Confidence interval; **RR:** Risk ratio;

GRADE Working Group grades of evidence
High quality: Further research is very unlikely to change our confidence in the estimate of effect.
Moderate quality: Further research is likely to have an important impact on our confidence in the estimate of effect and may change the estimate.
Low quality: Further research is very likely to have an important impact on our confidence in the estimate of effect and is likely to change the estimate.
Very low quality: We are very uncertain about the estimate.

SR in 6 months for primary immune thrombocytopenia

Patient or population: patients with primary immune thrombocytopenia
Settings:
Intervention: SR in 6 months

Outcomes	Illustrative comparative risks* (95% CI)		Relative effect (95% CI)	No of Participants (studies)	Quality of the evidence (GRADE)	Comments
	Assumed risk<br>Control	Corresponding risk<br>SR in 6 months				
SR in 6 months	**Study population**		RR 1.73 (1.36 to 2.19)	296 (3 studies)	⊕⊕⊕⊝ **moderate**[1]	
	**370 per 1000**	**640 per 1000** (503 to 810)				
	**Medium risk population**					
	**366 per 1000**	**633 per 1000** (498 to 802)				

*The basis for the **assumed risk** (e.g. the median control group risk across studies) is provided in footnotes. The **corresponding risk** (and its 95% confidence interval) is based on the assumed risk in the comparison group and the **relative effect** of the intervention (and its 95% CI).

CI: Confidence interval; **RR:** Risk ratio;

GRADE Working Group grades of evidence
High quality: Further research is very unlikely to change our confidence in the estimate of effect.
Moderate quality: Further research is likely to have an important impact on our confidence in the estimate of effect and may change the estimate.
Low quality: Further research is very likely to have an important impact on our confidence in the estimate of effect and is likely to change the estimate.
Very low quality: We are very uncertain about the estimate.

[1] concealment of allocation

SR in 12 months for primary immune thrombocytopenia

Patient or population: patients with primary immune thrombocytopenia
Settings:
Intervention: SR in 12 months

Outcomes	Illustrative comparative risks* (95% CI)		Relative effect (95% CI)	No of Participants (studies)	Quality of the evidence (GRADE)	Comments
	Assumed risk<br>Control	Corresponding risk<br>SR in 12 months				
SR in 12 months	**Study population**		RR 2.19 (1.6 to 3.02)	274 (3 studies)	⊕⊕⊕⊝ **moderate**[1]	
	**262 per 1000**	**574 per 1000** (419 to 791)				
	**Medium risk population**					
	**222 per 1000**	**486 per 1000** (355 to 670)				

*The basis for the **assumed risk** (e.g. the median control group risk across studies) is provided in footnotes. The **corresponding risk** (and its 95% confidence interval) is based on the assumed risk in the comparison group and the **relative effect** of the intervention (and its 95% CI).

CI: Confidence interval; **RR:** Risk ratio;

GRADE Working Group grades of evidence
High quality: Further research is very unlikely to change our confidence in the estimate of effect.
Moderate quality: Further research is likely to have an important impact on our confidence in the estimate of effect and may change the estimate.
Low quality: Further research is very likely to have an important impact on our confidence in the estimate of effect and is likely to change the estimate.
Very low quality: We are very uncertain about the estimate.

[1] concealment of allocation

Relapse for primary immune thrombocytopenia

Patient or population: patients with primary immune thrombocytopenia
Settings:
Intervention: Relapse

Outcomes	Illustrative comparative risks* (95% CI)		Relative effect (95% CI)	No of Participants (studies)	Quality of the evidence (GRADE)	Comments
	Assumed risk<br>Control	Corresponding risk<br>Relapse				
Relapse	**Study population**		RR 0.63 (0.4 to 1.02)	437 (5 studies)	⊕⊕⊕⊕ **high**	
	**269 per 1000**	**169 per 1000** (108 to 274)				
	**Medium risk population**					
	**254 per 1000**	**160 per 1000** (102 to 259)				

*The basis for the **assumed risk** (e.g. the median control group risk across studies) is provided in footnotes. The **corresponding risk** (and its 95% confidence interval) is based on the assumed risk in the comparison group and the **relative effect** of the intervention (and its 95% CI).

CI: Confidence interval; **RR:** Risk ratio;

GRADE Working Group grades of evidence
High quality: Further research is very unlikely to change our confidence in the estimate of effect.
Moderate quality: Further research is likely to have an important impact on our confidence in the estimate of effect and may change the estimate.
Low quality: Further research is very likely to have an important impact on our confidence in the estimate of effect and is likely to change the estimate.
Very low quality: We are very uncertain about the estimate.

Treg on baseline for primary immune thrombocytopenia

Patient or population: patients with primary immune thrombocytopenia
Settings:
Intervention: Treg on baseline

Outcomes	Illustrative comparative risks* (95% CI)		Relative effect (95% CI)	No of Participants (studies)	Quality of the evidence (GRADE)	Comments
	Assumed risk	Corresponding risk				
	**Control**	**Treg on baseline**				
**Treg on baseline**		The mean Treg on baseline in the intervention groups was **0 higher** (0.2 lower to 0.19 higher)		246 (3 studies)	⊕⊕⊕⊖ **moderate**[1]	

*The basis for the **assumed risk** (e.g. the median control group risk across studies) is provided in footnotes. The **corresponding risk** (and its 95% confidence interval) is based on the assumed risk in the comparison group and the **relative effect** of the intervention (and its 95% CI).

CI: Confidence interval;

GRADE Working Group grades of evidence
High quality: Further research is very unlikely to change our confidence in the estimate of effect.
Moderate quality: Further research is likely to have an important impact on our confidence in the estimate of effect and may change the estimate.
Low quality: Further research is very likely to have an important impact on our confidence in the estimate of effect and is likely to change the estimate.
Very low quality: We are very uncertain about the estimate.

[1] risk of bias

Treg day14 for primary immune thrombocytopenia

Patient or population: patients with primary immune thrombocytopenia
Settings:
Intervention: Treg day14

Outcomes	Illustrative comparative risks* (95% CI)		Relative effect (95% CI)	No of Participants (studies)	Quality of the evidence (GRADE)	Comments
	Assumed risk	Corresponding risk				
	**Control**	**Treg day14**				
**Treg day14**		The mean Treg day14 in the intervention groups was **1.02 higher** (0.76 to 1.28 higher)		246 (3 studies)	⊕⊕⊕⊖ **moderate**[1]	

*The basis for the **assumed risk** (e.g. the median control group risk across studies) is provided in footnotes. The **corresponding risk** (and its 95% confidence interval) is based on the assumed risk in the comparison group and the **relative effect** of the intervention (and its 95% CI).

CI: Confidence interval;

GRADE Working Group grades of evidence
High quality: Further research is very unlikely to change our confidence in the estimate of effect.
Moderate quality: Further research is likely to have an important impact on our confidence in the estimate of effect and may change the estimate.
Low quality: Further research is very likely to have an important impact on our confidence in the estimate of effect and is likely to change the estimate.
Very low quality: We are very uncertain about the estimate.

[1] risk of bias

Treg on day 28 for primary immune thrombocytopenia

Patient or population: patients with primary immune thrombocytopenia
Settings:
Intervention: Treg on day 28

Outcomes	Illustrative comparative risks* (95% CI)		Relative effect (95% CI)	No of Participants (studies)	Quality of the evidence (GRADE)	Comments
	Assumed risk	Corresponding risk				
	**Control**	**Treg on day 28**				
**Treg on day 28**		The mean Treg on day 28 in the intervention groups was **2.19 higher** (1.6 to 2.77 higher)		246 (3 studies)	⊕⊕⊕⊖ **moderate**[1]	

*The basis for the **assumed risk** (e.g. the median control group risk across studies) is provided in footnotes. The **corresponding risk** (and its 95% confidence interval) is based on the assumed risk in the comparison group and the **relative effect** of the intervention (and its 95% CI).

CI: Confidence interval;

GRADE Working Group grades of evidence
High quality: Further research is very unlikely to change our confidence in the estimate of effect.
Moderate quality: Further research is likely to have an important impact on our confidence in the estimate of effect and may change the estimate.
Low quality: Further research is very likely to have an important impact on our confidence in the estimate of effect and is likely to change the estimate.
Very low quality: We are very uncertain about the estimate.

[1] risk of bias

serious AE for primary immune thrombocytopenia

Patient or population: patients with primary immune thrombocytopenia
Settings:
Intervention: serious AE

Outcomes	Illustrative comparative risks* (95% CI)		Relative effect (95% CI)	No of Participants (studies)	Quality of the evidence (GRADE)	Comments
	Assumed risk	Corresponding risk				
	Control	Serious AE				
serious AE	**Study population**		RR 1.93 (1 to 3.71)	286 (3 studies)	⊕⊕⊕⊕ **high**	
	**81 per 1000**	**156 per 1000** (81 to 301)				
	**Medium risk population**					
	**80 per 1000**	**154 per 1000** (80 to 297)				

*The basis for the **assumed risk** (e.g. the median control group risk across studies) is provided in footnotes. The **corresponding risk** (and its 95% confidence interval) is based on the assumed risk in the comparison group and the **relative effect** of the intervention (and its 95% CI).

CI: Confidence interval; **RR:** Risk ratio;

GRADE Working Group grades of evidence
High quality: Further research is very unlikely to change our confidence in the estimate of effect.
Moderate quality: Further research is likely to have an important impact on our confidence in the estimate of effect and may change the estimate.
Low quality: Further research is very likely to have an important impact on our confidence in the estimate of effect and is likely to change the estimate.
Very low quality: We are very uncertain about the estimate.

infection for primary immune thrombocytopenia

Patient or population: patients with primary immune thrombocytopenia
Settings:
Intervention: infection

Outcomes	Illustrative comparative risks* (95% CI)		Relative effect (95% CI)	No of Participants (studies)	Quality of the evidence (GRADE)	Comments
	Assumed risk	Corresponding risk				
	Control	Infection				
infection	**Study population**		RR 1.19 (0.86 to 1.65)	684 (8 studies)	⊕⊕⊖⊖ **low**[1,2]	
	**135 per 1000**	**161 per 1000** (116 to 223)				
	**Medium risk population**					
	**44 per 1000**	**52 per 1000** (38 to 73)				

*The basis for the **assumed risk** (e.g. the median control group risk across studies) is provided in footnotes. The **corresponding risk** (and its 95% confidence interval) is based on the assumed risk in the comparison group and the **relative effect** of the intervention (and its 95% CI).

CI: Confidence interval; **RR:** Risk ratio;

GRADE Working Group grades of evidence
High quality: Further research is very unlikely to change our confidence in the estimate of effect.
Moderate quality: Further research is likely to have an important impact on our confidence in the estimate of effect and may change the estimate.
Low quality: Further research is very likely to have an important impact on our confidence in the estimate of effect and is likely to change the estimate.
Very low quality: We are very uncertain about the estimate.

[1] risk of bias
[2] publication bias exsisted

hyperglycemia for primary immune thrombocytopenia

Patient or population: patients with primary immune thrombocytopenia
Settings:
Intervention: hyperglycemia

Outcomes	Illustrative comparative risks* (95% CI)		Relative effect (95% CI)	No of Participants (studies)	Quality of the evidence (GRADE)	Comments
	Assumed risk	Corresponding risk				
	Control	Hyperglycemia				
hyperglycemia	**Study population**		RR 0.9 (0.58 to 1.38)	546 (7 studies)	⊕⊕⊕⊖ **moderate**[1]	
	**120 per 1000**	**108 per 1000** (70 to 166)				
	**Medium risk population**					
	**86 per 1000**	**77 per 1000** (50 to 119)				

*The basis for the **assumed risk** (e.g. the median control group risk across studies) is provided in footnotes. The **corresponding risk** (and its 95% confidence interval) is based on the assumed risk in the comparison group and the **relative effect** of the intervention (and its 95% CI).

CI: Confidence interval; **RR:** Risk ratio;

GRADE Working Group grades of evidence
High quality: Further research is very unlikely to change our confidence in the estimate of effect.
Moderate quality: Further research is likely to have an important impact on our confidence in the estimate of effect and may change the estimate.
Low quality: Further research is very likely to have an important impact on our confidence in the estimate of effect and is likely to change the estimate.
Very low quality: We are very uncertain about the estimate.

[1] risk os bias

hypertension for primary immune thrombocytopenia

Patient or population: patients with primary immune thrombocytopenia
Settings:
Intervention: hypertension

Outcomes	Illustrative comparative risks* (95% CI)		Relative effect (95% CI)	No of Participants (studies)	Quality of the evidence (GRADE)	Comments
	Assumed risk	Corresponding risk				
	Control	Hypertension				
hypertension	Study population		RR 1.19 (0.75 to 1.89)	367 (5 studies)	⊕⊕⊕⊖ moderate[1]	
	137 per 1000	163 per 1000 (103 to 259)				
	Medium risk population					
	57 per 1000	68 per 1000 (43 to 108)				

*The basis for the **assumed risk** (e.g. the median control group risk across studies) is provided in footnotes. The **corresponding risk** (and its 95% confidence interval) is based on the assumed risk in the comparison group and the **relative effect** of the intervention (and its 95% CI).

CI: Confidence interval; **RR:** Risk ratio;

GRADE Working Group grades of evidence
High quality: Further research is very unlikely to change our confidence in the estimate of effect.
Moderate quality: Further research is likely to have an important impact on our confidence in the estimate of effect and may change the estimate.
Low quality: Further research is very likely to have an important impact on our confidence in the estimate of effect and is likely to change the estimate.
Very low quality: We are very uncertain about the estimate.

[1] risk of bias

electrolyte disorder for primary immune thrombocytopenia

Patient or population: patients with primary immune thrombocytopenia
Settings:
Intervention: electrolyte disorder

Outcomes	Illustrative comparative risks* (95% CI)		Relative effect (95% CI)	No of Participants (studies)	Quality of the evidence (GRADE)	Comments
	Assumed risk	Corresponding risk				
	Control	Electrolyte disorder				
electrolyte disorder	Study population		RR 1.13 (0.83 to 1.54)	394 (5 studies)	⊕⊕⊕⊖ moderate	
	244 per 1000	276 per 1000 (203 to 376)				
	Medium risk population					
	80 per 1000	90 per 1000 (66 to 123)				

*The basis for the **assumed risk** (e.g. the median control group risk across studies) is provided in footnotes. The **corresponding risk** (and its 95% confidence interval) is based on the assumed risk in the comparison group and the **relative effect** of the intervention (and its 95% CI).

CI: Confidence interval; **RR:** Risk ratio;

GRADE Working Group grades of evidence
High quality: Further research is very unlikely to change our confidence in the estimate of effect.
Moderate quality: Further research is likely to have an important impact on our confidence in the estimate of effect and may change the estimate.
Low quality: Further research is very likely to have an important impact on our confidence in the estimate of effect and is likely to change the estimate.
Very low quality: We are very uncertain about the estimate.

fever for primary immune thrombocytopenia

Patient or population: patients with primary immune thrombocytopenia
Settings:
Intervention: fever

Outcomes	Illustrative comparative risks* (95% CI)		Relative effect (95% CI)	No of Participants (studies)	Quality of the evidence (GRADE)	Comments
	Assumed risk	Corresponding risk				
	Control	Fever				
fever	Study population		RR 4.3 (0.92 to 20.06)	290 (3 studies)	⊕⊕⊕⊕ high[1]	
	7 per 1000	30 per 1000 (6 to 140)				
	Medium risk population					
	0 per 1000	0 per 1000 (0 to 0)				

*The basis for the **assumed risk** (e.g. the median control group risk across studies) is provided in footnotes. The **corresponding risk** (and its 95% confidence interval) is based on the assumed risk in the comparison group and the **relative effect** of the intervention (and its 95% CI).

CI: Confidence interval; **RR:** Risk ratio;

GRADE Working Group grades of evidence
High quality: Further research is very unlikely to change our confidence in the estimate of effect.
Moderate quality: Further research is likely to have an important impact on our confidence in the estimate of effect and may change the estimate.
Low quality: Further research is very likely to have an important impact on our confidence in the estimate of effect and is likely to change the estimate.
Very low quality: We are very uncertain about the estimate.

[1] risk of bias

erythra for primary immune thrombocytopenia

Patient or population: patients with primary immune thrombocytopenia
Settings:
Intervention: erythra

Outcomes	Illustrative comparative risks* (95% CI)		Relative effect (95% CI)	No of Participants (studies)	Quality of the evidence (GRADE)	Comments
	Assumed risk<br>**Control**	Corresponding risk<br>**Erythra**				
**erythra**	**Study population**		**RR 2.05**<br>(0.53 to 7.98)	246<br>(3 studies)	⊕⊕⊖⊖<br>**low**[1,2]	
	**16 per 1000**	**33 per 1000**<br>(8 to 128)				
	**Medium risk population**					
	**0 per 1000**	**0 per 1000**<br>(0 to 0)				

*The basis for the **assumed risk** (e.g. the median control group risk across studies) is provided in footnotes. The **corresponding risk** (and its 95% confidence interval) is based on the assumed risk in the comparison group and the **relative effect** of the intervention (and its 95% CI).

CI: Confidence interval; **RR:** Risk ratio;

GRADE Working Group grades of evidence
High quality: Further research is very unlikely to change our confidence in the estimate of effect.
Moderate quality: Further research is likely to have an important impact on our confidence in the estimate of effect and may change the estimate.
Low quality: Further research is very likely to have an important impact on our confidence in the estimate of effect and is likely to change the estimate.
Very low quality: We are very uncertain about the estimate.

[1] risk of bias
[2] publication bias exsisted

S2 Fig. Assessment of evidences by GRADE pro software.
