# Supplementary figures and images for: Efficacy and Safety of the Combination Treatment of Rituximab and Dexamethasone for Adults with Primary Immune Thrombocytopenia (ITP): A Meta-Analysis
Source: Biomed Res Int. 2018 Dec 12;2018:1316096. doi: 10.1155/2018/1316096 (PMC6311778; doi:10.1155/2018/1316096)

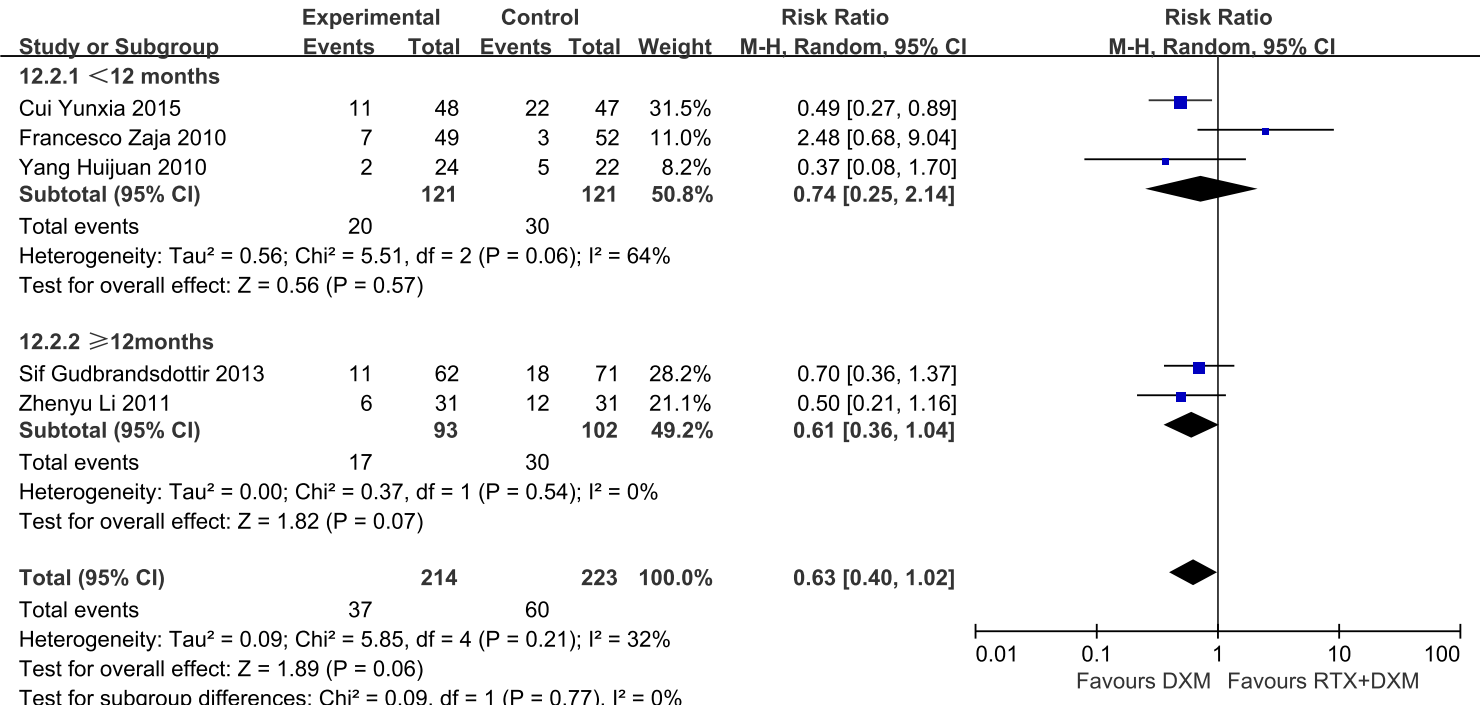

Supplement: Supplementary 3 — S3 Figure. Forest plots of relative risk in relapse rate. [file 1316096.f3.pdf]

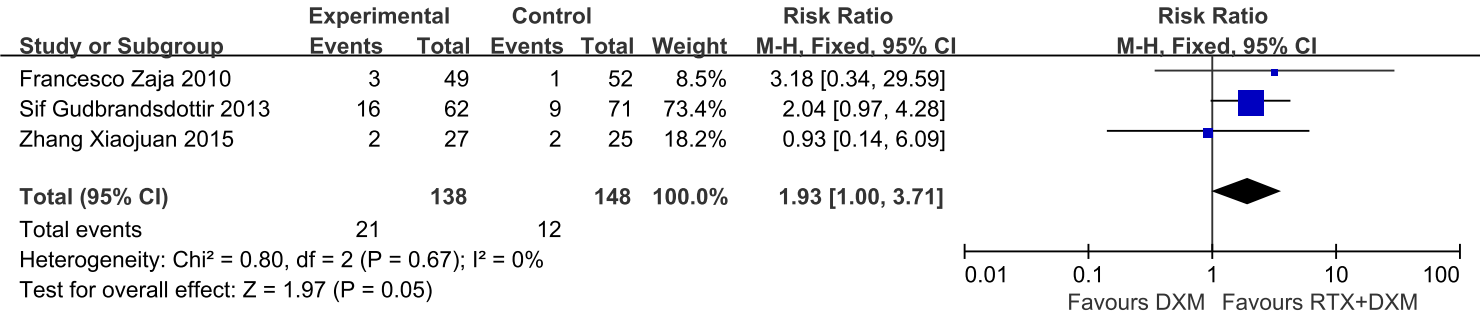

Supplement: Supplementary 4 — S4 Figure. Forest plots of relative risk in serious adverse effects. [file 1316096.f4.pdf]
